# Adaptive Amplification and Point Mutation Are Independent Mechanisms: Evidence for Various Stress-Inducible Mutation Mechanisms

**DOI:** 10.1371/journal.pbio.0020399

**Published:** 2004-11-23

**Authors:** P. J Hastings, Andrew Slack, Joseph F Petrosino, Susan M Rosenberg

**Affiliations:** **1**Department of Molecular and Human Genetics, Baylor College of MedicineHouston, TexasUnited States of America; **2**Department of Biochemistry and Molecular Biology and Department of Molecular Virology and Microbiology, Baylor College of MedicineHouston, TexasUnited States of America

## Abstract

“Adaptive mutation” denotes a collection of processes in which cells respond to growth-limiting environments by producing compensatory mutants that grow well, apparently violating fundamental principles of evolution. In a well-studied model, starvation of stationary-phase *lac^−^ Escherichia coli* cells on lactose medium induces Lac^+^ revertants at higher frequencies than predicted by usual mutation models. These revertants carry either a compensatory frameshift mutation or a greater than 20-fold amplification of the leaky *lac* allele. A crucial distinction between alternative hypotheses for the mechanisms of adaptive mutation hinges on whether these amplification and frameshift mutation events are distinct, or whether amplification is a molecular intermediate, producing an intermediate cell type, in colonies on a pathway to frameshift mutation. The latter model allows the evolutionarily conservative idea of increased mutations (per cell) without increased mutation rate (by virtue of extra gene copies per cell), whereas the former requires an increase in mutation rate, potentially accelerating evolution. To resolve these models, we probed early events leading to rare adaptive mutations and report several results that show that amplification is not the precursor to frameshift mutation but rather is an independent adaptive outcome. (i) Using new high-resolution selection methods and stringent analysis of all cells in very young (micro)colonies (500–10,000 cells), we find that most mutant colonies contain no detectable *lac*-amplified cells, in contrast with previous reports. (ii) Analysis of nascent colonies, as young as the two-cell stage, revealed mutant Lac^+^ cells with no *lac-*amplified cells present. (iii) Stringent colony-fate experiments show that microcolonies of *lac*-amplified cells grow to form visible colonies of *lac*-amplified, not mutant, cells. (iv) Mutant cells do not overgrow *lac*-amplified cells in microcolonies fast enough to mask the *lac*-amplified cells. (v) *lac*-amplified cells are not SOS-induced, as was proposed to explain elevated mutation in a sequential model. (vi) Amplification, and not frameshift mutation, requires DNA polymerase I, demonstrating that mutation is separable from amplification, and also illuminating the amplification mechanism. We conclude that amplification and mutation are independent outcomes of adaptive genetic change. We suggest that the availability of alternative pathways for genetic/evolutionary adaptation and clonal expansion under stress may be exploited during processes ranging from the evolution of drug resistance to cancer progression.

## Abstract 2

Cells can respond to stress by apparently increasing their mutation rate. This study provides evidence that there is more than one pathway by which cells achieve such a response

## Introduction

Adaptive mutation was first brought to wide attention by Cairns et al. (1988), and encompasses a collection of processes whereby cells adapt genetically in response to growth-limiting environments. The phenomenon is reported in several different microbial systems, and appears to occur via different molecular mechanisms (reviewed by Rosenberg 2001; Hersh et al. 2004). The Lac frameshift assay system of Escherichia coli (Cairns and Foster 1991) is the best understood in terms of mutation mechanism (reviewed by Foster 1999; Rosenberg 2001; Hersh et al. 2004). In this system, cells carrying a +1 frameshift mutation in *lac* genes on an F′ conjugative plasmid are starved on solid medium with lactose as the sole carbon source, selecting Lac^+^ revertants. Lac^+^ clones appear over time during selection in a population of cells that shows no net growth (i.e., is in stationary phase). In most clones, Lac^+^ is conferred by a compensatory −1 frameshift mutation in the *lac* gene (Lac^+^ point mutants). Lac^+^ point mutation during stationary phase differs from mutation during rapid growth in that it requires the recombination proteins RecA, RecBC, RuvA, RuvB, and RuvC (Harris et al. 1994, 1996; Foster et al. 1996), and induction of the SOS DNA damage response regulon (Cairns and Foster 1991; McKenzie et al. 2000), particularly error-prone DNA polymerase (Pol) IV (DinB) (McKenzie et al. 2001, 2003; Wolff et al. 2004). The *lac* frameshift allele is leaky, conferring 1%–2% of the wild-type level of β-galactosidase (Foster 1994; Andersson et al. 1998), and Lac^+^ colonies can also occur by amplification of the *lac* locus into a tandem array of 20–100 copies (Foster 1994; Andersson et al. 1998; Hastings et al. 2000). Adaptive amplification requires RpoS, the transcriptional activator of approximately 50 stationary-phase/starvation- and general-stress-response-specific genes, which is also required for point mutation (Lombardo et al. 2004). Amplification does not require DinB or induction of other SOS proteins (McKenzie et al. 2001), and whether or not it requires recombination proteins has not been determined unambiguously (Tlsty et al. 1984; Hastings and Rosenberg 2002).

Consistent with Darwinian principles of evolution by selection of undirected and random genetic changes, adaptive point mutation in the Lac system is accompanied by a high level of mutation in genes unrelated to lactose catabolism (secondary mutation). Lac^+^ point mutants show about 50-fold more secondary mutations than do cells starved on the same plate that did not become Lac^+^ (Torkelson et al. 1997; Rosche and Foster 1999; Godoy et al. 2000). This indicates that a subpopulation of the starved cells experiences a transient episode of hypermutation (adaptive point mutants are not mutator mutants; Torkelson et al. 1997; Rosenberg et al. 1998; Rosche and Foster 1999). In keeping with their independence of DinB and SOS, the amplified clones do not show hypermutation of unrelated genes (Hastings et al. 2000). These differences, together with the finding that cells carrying *lac* amplification (“*lac*-amplified cells”) do not readily yield Lac^+^ point mutants when resubjected to selection on lactose medium, led us to propose that amplification reflects a pathway of genetic change wholly or partly separate from point mutation (we suggested an alternative outcome of a branched pathway; Hastings et al. 2000; Rosenberg 2001; Hastings and Rosenberg 2002).

Two general models for the adaptive point mutation mechanism in this system are currently at odds, each focused on the role of DNA amplification, and each representing a different view of evolution (e.g., Rosenberg and Hastings 2004a, 2004b; Roth and Andersson 2004a, 2004b). In “hypermutation models,” the observed high frequency of Lac^+^ point mutants is proposed to result from transient hypermutation as part of a stress response, one consequence of which is acceleration of genetic change (and so potentially of evolution) (e.g., see Rosenberg 2001, and below, where specific molecular mechanisms are suggested). In this view, amplification is an adaptive pathway alternative to point mutation that allows growth of cells, relieving the stress of starvation, and so deflects them from the point mutation route. This view is compatible with Darwinism, which allows for changing rates of generation of heritable variations (Darwin 1859).

In an alternative model, called “amplification–mutagenesis,” amplification is an intermediate in the formation of Lac^+^ point mutants, and its most important role is to provide more *lac* copies per cell so that point mutation can occur without increase in mutation rate per copy of *lac* (Andersson et al. 1998; Hendrickson et al. 2002). This adheres to conservative neo-Darwinist ideas of constant and gradual evolutionary change (e.g., Mayr 1982).

Here, we report several experiments that demonstrate that amplification and point mutation are separate adaptive outcomes, compatible with hypermutation models, and not part of a sequential pathway leading to mutation (as in the amplification–mutagenesis model). We also report the first genetic requirement specific to the adaptive amplification pathway, Pol I.

## Results/Discussion

### Colony Phenotypes

**Figure 1:**
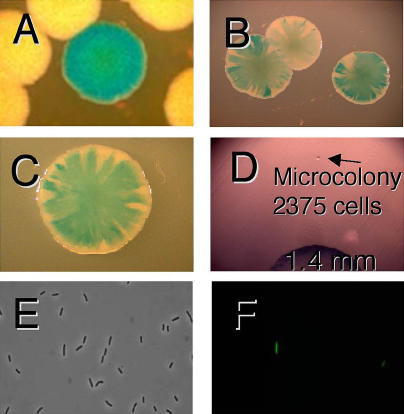
Colony Morphologies (A) Point-mutant Lac^+^ colony showing solid blue color (the pale colonies are derived from Lac^−^ cells). (B) *lac*-amplified colonies showing sectoring caused by the instability of the amplified array. Cells from these colonies grow either into sectored blue colonies or, if they have lost the amplification, into white colonies, a phenotype that we call “unstable.” (C) A sectored colony that is not unstable in that it was found to contain only stable blue and stable white cfu upon retesting. (D) An example of a microcolony of the sort used in this work. The visible colony on the lower edge of the field has a diameter of 1.4 mm (>10^8^ cells). (E and F) Phase contrast (E) and green fluorescence (F) of the same field, showing two of 30 SMR6039 cells fluorescing.

Experiments described below make use of the different colony color phenotypes of point-mutant and *lac*-amplified clones grown on rich medium with the dye 5-bromo-4-chloro-3-indoyl β-D-galactoside (X-gal) (e.g., Tlsty et al. 1984; Andersson et al. 1998; Hastings et al. 2000) (Figure 1). We use the word “sectored” to describe the appearance of a colony of cells on X-gal medium (e.g., Figure 1B and 1C). The word “unstable” is used to denote continued sectoring when cells from sectored colonies are replated. This instability has been shown in many examples to reflect tandem amplification of the *lac* region (Tlsty et al. 1984; Foster 1994; Andersson et al. 1998; Hastings et al. 2000). Sectoring that is not unstable (Figure 1C) may reflect incidental juxtaposition of blue and white colonies on a plate (see below). “Stable” describes the absence of visible sectoring that indicates Lac^+^ point mutation (Tlsty et al. 1984; Hastings et al. 2000; see Figure 1A).

### Most Microcolonies Are Pure, Not Mixed

**Figure 2:**
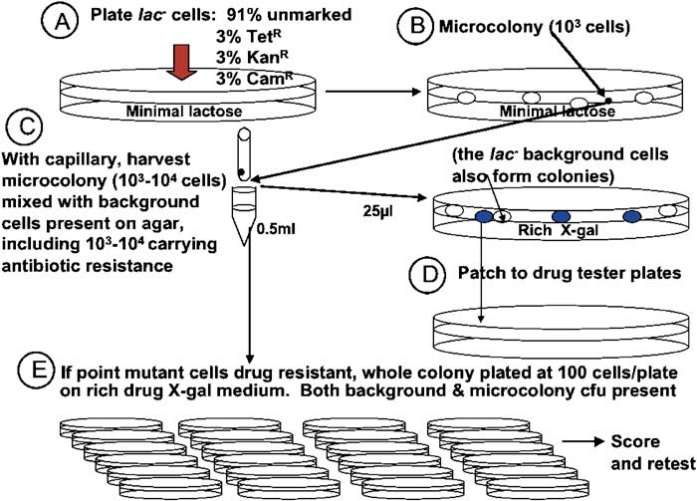
Stringent Analysis of Whole Microcolonies: Analyzing All Cells in a Microcolony and Reducing Contamination by Unrelated Neighboring Bacteria To analyze all cells in a microcolony, very young microcolonies were harvested (10^3^–10^4^ cfu/microcolony; see Figure 1D; Table 1). To reduce contamination with neighboring Lac^−^ bacteria and unrelated Lac^+^ microcolonies from the minimal-lactose selection plate, first, a minority of the Lac^−^ cells plated carried an antibiotic-resistance marker, and only resistant microcolonies were analyzed, and, second, sectored colonies observed were retested for instability (to eliminate sectored colonies that are not unstable, shown in Figure 1C, which may result from accidental overlap of blue and white cfu). Procedures described in Materials and Methods, “whole microcolony analysis.” Cam^R^, chloramphenicol resistant; Kan^R^, kanamycin resistant; Tet^R^, tetracycline resistant. Streptomycin resistance (not shown) was also used.

If most adaptive point mutants arise by mutation in young colonies carrying *lac* amplification (Andersson et al. 1998; Hendrickson et al. 2002), then young colonies should contain both the *lac*-amplified progenitor cells and their point-mutant descendants, as reported by Hendrickson et al. (2002) and reexamined here. To determine whether a microcolony carries point-mutant or *lac-*amplified cells or both, in principle, one picks the microcolony from the lactose-minimal selection medium and replates its cells onto rich X-gal medium to observe whether the resulting colonies are pure blue (point mutant), unstable sectoring (*lac-*amplified), or both. A significant problem in interpreting data from such analyses is that the picked microcolony carries with it many Lac^−^ background cells from the selection plate (Figure 2C) and may contain cells from unrelated microcolonies, because, of necessity, these microcolonies cannot be streaked to purify them before analysis.

**Table 1:**
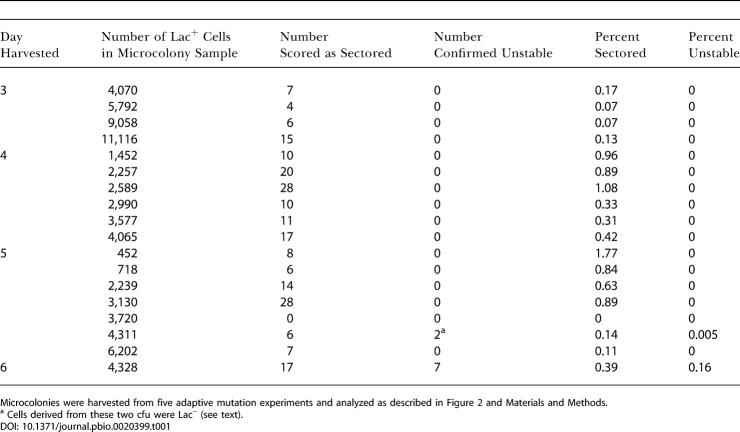
Composition of Whole Microcolonies

Microcolonies were harvested from five adaptive mutation experiments and analyzed as described in Figure 2 and Materials and Methods

^a^ Cells derived from these two cfu were Lac^−^ (see text)

10.1371/journal.pbio.0020399.t001

We have improved the sensitivity of microcolony analysis substantially, first, by analyzing all cells in a microcolony (rather than a sample) and, second, by imposing stringent methods to reduce contamination of microcolonies with neighboring cells and microcolonies from the selection plate (Materials and Methods; Figure 2). First, frameshift-bearing (tester) cells were mixed with 3% each of three or four different derivative testers that carry antibiotic resistance markers (Figure 2A), and only microcolonies that carried one of these minority antibiotic resistance markers were analyzed to search for sectored colonies on medium carrying the antibiotic. This reduces, but does not eliminate, accidental overlap with Lac^−^ background cells and with unrelated Lac^+^ microcolonies accidentally harvested in the same sample (shown below). Second, because some sectored colonies are not unstable (e.g., mixtures of stable, white Lac^−^ background cells, and blue colony formers that overlapped), all sectored colonies found were retested by plating a sample on the same X-gal antibiotic medium to determine whether there was continued instability indicative of amplification (Figure 2E). Third, because the sequential model predicts that the younger the microcolony is, the greater the proportion of *lac*-amplified cells (colony-forming units [cfu]) it should contain (Hendrickson et al. 2002), and because one might miss the putative *lac-*amplified cfu by not examining enough cfu per microcolony, we analyzed very young microcolonies of 10^2^–10^4^ cfu/microcolony (as compared with approximately 10^5^ cfu/microcolony used by Hendrickson et al. [2002]) and analyzed *all* cfu in each of them (rather than a sample) (Figure 2 Table 1).

The results of analyzing every cell in 18 whole microcolonies of stable cells, isolated on days 3 through 6 of adaptive mutation experiments, are shown in Table 1. Although we found sectored colonies at frequencies of 10^−2^–10^−3^ in almost all microcolonies (as reported by Hendrickson et al. 2002), these sectored colonies, when replated, were almost all found to consist of cells giving rise to stable white and stable blue colonies only, indicating that these were not from *lac*-amplified clones (also demonstrated below). In general, we looked at 50 to 100 cells from these replated sectored colonies, but, for three of them, we screened 3,000–4,000 cells with the same result: we could find no unstable cfu within them. In contrast, cells from *lac*-amplified colonies, upon replating, again give rise to sectored colonies. Therefore, most of the sectored colonies that we found give no evidence of carrying amplification of the *lac* locus. Sectoring in these colonies may occur by chance juxtaposition of Lac^+^ point-mutant cells with non-mutated background cells (which are numerous) during the plating (Figure 2). Other explanations might also be possible; however, these colonies are not *lac-*amplification bearers by this test and also as demonstrated in the following section. An example of such a colony is shown in Figure 1C. Such colonies may account for the report of Hendrickson et al. (2002) that more than 10^−3^ of cells in every Lac^+^ microcolony are sectored colony formers. These sectored, non-*lac*-amplified colonies have also been reported by another laboratory (Poteete et al. 2002).

Of the 18 whole microcolonies examined (Table 1), 16 were pure stable. One day-6 microcolony was found to contain seven unstable cfu. (Another, found on day 5, contained two cfu showing sectors of an extremely pale blue color along with sectors of the same color as the Lac^−^ parental cells.) Those unstable cfu might be clonally related to the rest of the microcolony, or might result from chance juxtaposition of two different clones carrying the same antibiotic resistance marker. To determine the frequency at which such chance juxtapositions are expected, we analyzed the cells carried on the agar plugs on which microcolonies were harvested. We did this by plating the microcolony suspensions on medium containing an antibiotic to which cells of the microcolony were sensitive, to ascertain the frequency of marked Lac^+^ cells that are unrelated to a microcolony but get harvested with it. The microcolony plugs contained from 900 to 7,000 antibiotic-resistant *lac* ^−^ background cells each. Of eleven such plugs analyzed, one contained three unrelated (different antibiotic resistance) point-mutant Lac^+^ cfu, one contained one unrelated unstable cfu, and three contained unrelated cfu of the very pale sectored phenotype described above. The presence on agar plugs of cells giving rise to sectored colonies that are unrelated to the microcolony, but occur at a frequency comparable to the frequency of unstables in a microcolony of the same antibiotic-resistance genotype (Table 1, last line), implies that the presence of unstable cfu was independent of the microcolony, and thus not relevant to the origin of the microcolony.

The very pale sectored colonies that we found were examined further and were shown to be unstable upon retesting but contained no cells capable of forming colonies on lactose medium under the conditions of an adaptive mutation experiment. Presumably they contain too few copies of the *lac* region to allow growth on lactose medium. They might carry *lac* gene duplication, or tandem amplification at a very low level. In support of this possibility, we note that their pale appearance on X-gal medium is similar to known *lac* gene duplications of this frameshift allele that we have constructed (A. Slack and P. J. Hastings, unpublished data). Another possibility is that they contain multiple F′ plasmids (Foster and Rosche 1999). Their failure to form adaptive point-mutant or *lac*-amplified colonies shows that they do not advance to a Lac^+^ phenotype efficiently (so are not obvious precursors to point mutation).

Thus, colonies composed of a mixture of *lac*-amplified and point-mutant cells, as reported by Andersson et al. (1998) and Hendrickson et al. (2002), are not common when stringent methods of scoring are imposed, and such mixed colonies are certainly not every microcolony in which a mutation has occurred. Those apparent mixed colonies that do occur are likely to be unrelated mixtures and not relevant to the origin of the Lac^+^ point mutants in the colony.

### Selection for *lac-*Amplified Cells in Point-Mutant Microcolonies

As an independent test for the presence of rare *lac-*amplified cells in point-mutant microcolonies, we devised a genetic selection capable of revealing single *lac*-amplified cells among numerous non-amplified cells. The selection depends on the quantitative relationship between the number of copies of the chloramphenicol acetyl transferase gene *(cat)* and the concentration of the antibiotic chloramphenicol that cells resist (Petit et al. 1992). We inserted the *cat* gene into *codA,* about 5 kb from *lac.* Control experiments established that strains with *lac* and *cat* amplification (as shown by unstable phenotypes and Southern blot analyses) could grow on rich Luria-Bertani-Herskowitz medium (LBH) containing 100 μg/ml chloramphenicol, whereas Lac^+^ point-mutant cells, and those amplified at *lac* but not at *cat,* did not form colonies at this concentration. We determined that 61% or more of all *lac*-amplified cells are detected by this selection (Materials and Methods).

The data are shown in Table 2. Among 75 Lac^+^ point-mutant microcolonies isolated from days 4, 5, and 6 of an adaptive mutation experiment, three (4%) were found to contain a few chloramphenicol-resistant (CamR) cfu (Table 2). All of these showed the *lac-*amplified colony phenotype. Correction for those that would not be detected by this selection (above; Materials and Methods) shows that, at most, 6.6% of point-mutant microcolonies contain any *lac*-amplified cells (and probably fewer; see below)*.* As discussed above, this contrasts with the report of Hendrickson et al. (2002) that all Lac^+^ microcolonies contain *lac*-amplified cells. Also, these data verify independently that the high levels of sectored, but not unstable, cfu found among microcolonies (see Table 1) were not *lac*-amplification bearers, because most *lac-*amplified cfu would have scored positively in this selection.

**Table 2:**
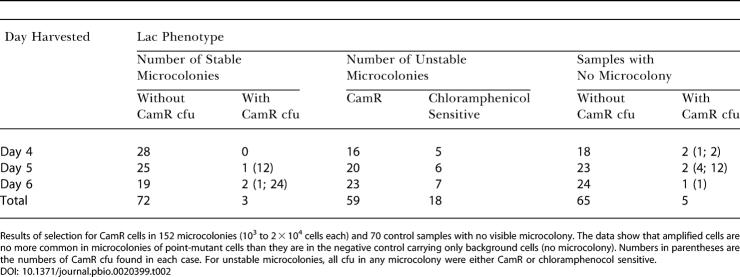
Selection for Rare Amplified Cells in Colonies of Point-Mutant Cells

Results of selection for CamR cells in 152 microcolonies (10^3^ to 2 × 10^4^ cells each) and 70 control samples with no visible microcolony. The data show that amplified cells are no more common in microcolonies of point-mutant cells than they are in the negative control carrying only background cells (no microcolony). Numbers in parentheses are the numbers of CamR cfu found in each case. For unstable microcolonies, all cfu in any microcolony were either CamR or chloramphenocol sensitive

10.1371/journal.pbio.0020399.t002

A negative control was performed by plating samples of cells from agar plugs that did not carry a microcolony visible on a dissecting microscope. Five of 70 such samples yielded one or a few cells that were *lac*-amplified and showed CamR. The similarity of this proportion to that for *lac*-amplified cells in point-mutant microcolonies suggests that none of the mixtures detected represents an occurrence of *lac*-amplified cells that arose in the same cell clone as the point mutation. Thus, we conclude that few, if any, point-mutant microcolonies include *lac*-amplified cells.

### Nascent Lac^+^ Point-Mutant Colonies Do Not Contain *lac*-Amplified Cells

The selection method described above allowed us to detect amplification in samples of stressed cells that did not include a visible microcolony. This suggested the possibility of analyzing point mutation and amplification in nascent Lac^+^ colonies as young as the two-cell stage and up to about 40 cells. The sequential amplification–mutagenesis model demands that all colonies as young as 2–40 cells must consist only of *lac-*amplified cells, because these are proposed not to generate Lac^+^ point mutations until the number of *lac* copies reaches 10^7^ or more (e.g., a colony of 10^5^ cells with 100 *lac* copies per cell caused by amplification; Hendrickson et al. 2002).

Cells from 600 sample plugs, harvested on days 3 to 5 in two experiments, that carried no microcolony visible at 20-fold magnification were analyzed by selecting for CamR in half the sample, and Lac^+^ phenotype in the other half. The samples were found to contain 1 × 10^4^ to 5 × 10^4^ total cells. We detected 22 events by either Lac^+^ or CamR selection (3.7% of the samples). This frequency of events is compatible with the observation of 30–60 Lac^+^ colonies per 10^8^ cells per day, which we see routinely in adaptive mutation experiments (e.g., Hastings et al. 2000). These events are listed in Table 3.

**Table 3:**
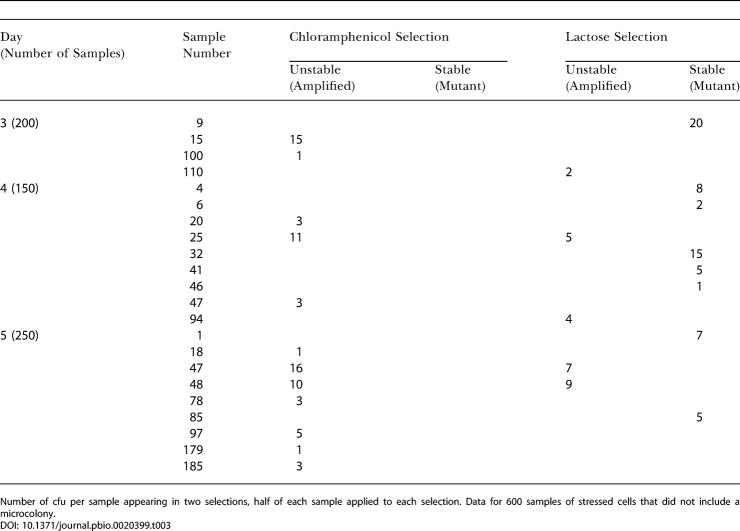
Nascent Lac^+^ Point-Mutant Colonies at the Few-Cell Stage Do Not Include Amplified Cells

Number of cfu per sample appearing in two selections, half of each sample applied to each selection. Data for 600 samples of stressed cells that did not include a microcolony

10.1371/journal.pbio.0020399.t003

Twelve samples gave *lac-*amplified cells (cfu) detected as CamR. The cell numbers ranged from one to 16 cells (from half the sample). Two amplification events gave Lac^+^ cells that were chloramphenicol sensitive, detected on lactose-minimal medium plates. Many more amplification events were detected by CamR selection than by growth on lactose medium (even though all were *lac-*amplified because of their being selected on lactose medium), implying that the CamR test is more sensitive, and capable of detecting levels of amplification that are too low to allow colony formation on lactose medium.

Eight samples gave stable Lac^+^ cells (cfu), ranging in number from one to 20 cells from half the sample (implying colonies ranging in size from 2–40 cells). None of these eight contained amplified cells, as determined by CamR, or by rescoring the lactose plates after 7 d, by which time any pre-existing *lac-*amplified cells would have formed colonies (Hastings et al. 2000). Thus, we see that, even at the 2- to 40-cell stage of colony growth, we are unable to detect a correlation between mutation and amplification events, and we conclude that these are independent processes. These results are incompatible with the idea that Lac^+^ adaptive point mutants result from point mutation events in young colonies of *lac*-amplified cells (Andersson et al. 1998; Hendrickson et al. 2002).

### *lac-*Amplified Microcolonies Arise Later than Point-Mutant Microcolonies

**Figure 3:**
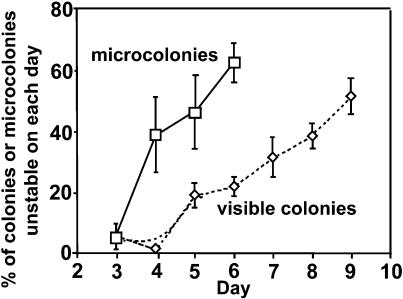
Temporal Distribution of *lac*-Amplified Microcolonies Data from six adaptive mutation microcolony experiments were pooled to give the distribution of point-mutant and *lac*-amplified phenotypes. Squares, the mean percentage of Lac^+^ microcolonies with unstable phenotype (± SEM; *n* = 3 experiments on day 3 and *n* = 4 on days 4, 5, and 6); diamonds, the percentage of *lac*-amplified visible colonies on the same days included for comparison (data from Hastings et al. 2000). Note that the observed proportion of unstable microcolonies is higher than the actual proportion because *lac*-amplified clones are slower growing than point mutants (Hastings et al. 2000), and so spend more time as microcolonies before becoming visible colonies. Thus, the lack of unstable microcolonies on early days is even more severe than the data show.

If point-mutant colonies arose from *lac*-amplified microcolonies, the majority of microcolonies of sufficiently small size would be expected to consist of *lac*-amplified cells. For example, in the sequential model of Hendrickson et al. (2002), Lac^+^ point mutation is expected to occur when a colony of *lac*-amplified cells grows to 10^5^ cells (with a mutation rate of 10^−8^ mutations per cell per generation that is enhanced 35-fold by SOS induction and acts on an additional 30 copies of *lac;* 35 × 30 × 10^5^ × 10^−8^ = 1 mutation per microcolony of 10^5^ cells). This model predicts that *lac*-amplified microcolonies of approximately 10^5^ cells should precede point-mutant colonies. As shown in Figure 3, the microcolonies harvested in the course of these experiments, all much smaller than 10^5^ cells, show a very distinct pattern that conflicts with this expectation. Only 5% of microcolonies isolated on day 3 consisted of *lac*-amplified cells (the rest were stable point mutant). The proportion of unstable microcolonies increases on later days. This is the pattern of arising of *lac*-amplified visible colonies, which are rare in the first few days of an experiment and increase in frequency later (Hastings et al. 2000; Figure 3). The proportion of unstable microcolonies appears to reflect the proportion of visible colonies that would consist of *lac*-amplified cells 1 to 4 d later (Figure 3), as expected if *lac*-amplified microcolonies produce *lac*-amplified (not point-mutant) visible colonies.

### *lac*-Amplified Microcolonies Produce *lac*-Amplified, Not Point-Mutant, Visible Colonies

**Figure 4:**
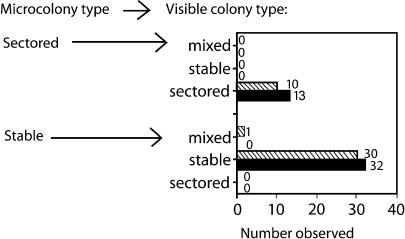
Fate of Microcolonies Developing In Situ Microcolonies on days 4 and 5 of separate adaptive mutation experiments were sampled by touching with a needle, then the sample was spread on rich X-gal medium and scored for point-mutant or sectored Lac^+^ phenotype (see Materials and Methods). The microcolonies were then left to grow into visible colonies (∼10^7^ cells), and the colonies sampled again and samples plated to score for sectoring (hatched bars, day 4 microcolony samples; black bars, day 5 microcolony samples). The numbers of each type of colony observed are shown next to the bars. One single stable microcolony produced a mixed visible colony, which may have been caused by overlap with another colony or microcolony.

The sequential amplification–mutagenesis model states that mutation occurs in a cell in a microcolony of *lac*-amplified cells (Hendrickson et al. 2002). Growth of the point-mutant cell clone is proposed to overtake the *lac*-amplified cells to give a visible colony consisting mainly of point-mutant cells. We looked for this change from *lac*-amplified to point-mutant phenotype by removing a few cells from microcolonies to determine which microcolonies consisted of *lac*-amplified cells, and sampling them again after they had grown to form visible colonies (Materials and Methods). The data in Figure 4 show that we did not see this change occur. *lac*-amplified microcolonies grew into *lac*-amplified visible colonies, and point-mutant microcolonies remained point mutant. We conclude that microcolonies of *lac*-amplified cells do not become visible colonies of point-mutant cells.

Hendrickson et al. (2002) reported a seemingly similar experiment that gave the opposite result. They scored small (presumed young) and large (old) colonies and found many more unstable cfu in small colonies than in larger colonies. We note that they did not follow the same small colonies to score later, when those small colonies had grown larger, as we have done. Their result is probably caused by the slow growth rate of *lac*-amplified cells, which causes microcolonies of *lac-*amplified cells to remain at the microcolony size for longer than do microcolonies of point-mutant cells, such that many more small colonies are *lac*-amplified. We have found that point-mutant microcolonies stay small for a much briefer period of time than do *lac*-amplified ones (data not shown). We overcame this problem by scoring *the same* colonies as microcolonies and found that these small *lac*-amplified colonies do not grow to become point mutant (Figure 4). An additional explanation for why the small colonies of Hendrickson et al. (2002) contained more *lac-*amplified cfu is that, in most of their experiments, these authors did not employ the stringent methods that we devised to avoid scoring unrelated mixtures of cells in microcolony samples. These include the antibiotic-resistant-minority-scoring method outlined in Figure 2. Thus, when they picked small colonies in a fixed volume of agar, a greater fraction of the cells in their sample would be unrelated background cells than when they picked larger colonies (same number of background cells each time, but fewer or more cells from the colony they intended to sample). This, too, should result in the appearance of more mixtures of *lac-*amplified and point-mutant cfu in the small colony samples and more pure point mutant in the large colony samples; but the mixtures in the small colony samples would be unrelated. We conclude that when stringent colony-fate experiments are performed, *lac-*amplified microcolonies are seen to produce *lac*-amplified and not point-mutant visible colonies (Figure 4).

### Point Mutants Do Not Overgrow *lac*-Amplified Cells in Competition Experiments

We tested experimentally whether point-mutant cells occurring in a *lac*-amplified microcolony would be able to overgrow and mask the *lac*-amplified cells on the timescale of adaptive mutation experiments, as is postulated in sequential models (Hendrickson et al. 2002). Cultures of *lac*-amplified cells were seeded with Lac^+^ point-mutant cells in various proportions and grown for a known number of cell generations, as spots on lactose plates, to form pseudocolonies. The proportion of *lac*-amplified and point-mutant cells was determined after 3 d, by which time the pseudocolony had reached 8 × 10^7^ to 1.6 × 10^8^ cells (Table 4). This is three to four more generations than are needed to form a visible colony (approximately 0.5–1 × 10^7^ cells; Hastings et al. 2000). These experiments were performed with two different *lac*-amplified isolates that have 2.0 and 4.2 times the generation time on lactose medium of the point-mutant Lac^+^ strain used. That is, these strains can produce 2 and 4.2 times fewer generations than point mutants, and so might, in principle, be overtaken by point mutants during colony growth. However, this was not observed under the real-life conditions of growth in colonies under selective conditions. Only when point-mutant cells were introduced at a ratio of one to ten was the proportion of *lac*-amplified cells reduced to below 1% (the level reported in mixed colonies by Hendrickson et al. [2002]), and then only with the slower growing *lac*-amplified isolate (Table 4). We did not see the point-mutant cells take over when the point mutant started at 10^−5^ of the cells, the predicted time of occurrence of a mutation (Hendrickson et al. 2002). Similar results were obtained when the experiment was performed in liquid culture (data not shown).

**Table 4:**
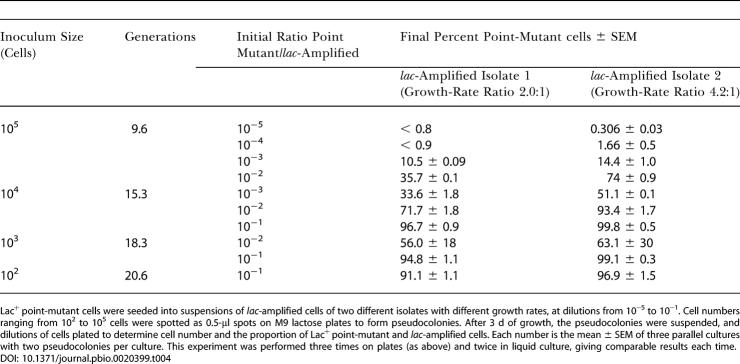
Competition between Two *lac*-Amplified Isolates and a Point-Mutant Lac^+^ Strain

Lac^+^ point-mutant cells were seeded into suspensions of *lac*-amplified cells of two different isolates with different growth rates, at dilutions from 10^−5^ to 10^−1^. Cell numbers ranging from 10^2^ to 10^5^ cells were spotted as 0.5-μl spots on M9 lactose plates to form pseudocolonies. After 3 d of growth, the pseudocolonies were suspended, and dilutions of cells plated to determine cell number and the proportion of Lac^+^ point-mutant and *lac*-amplified cells. Each number is the mean ± SEM of three parallel cultures with two pseudocolonies per culture. This experiment was performed three times on plates (as above) and twice in liquid culture, giving comparable results each time

10.1371/journal.pbio.0020399.t004

We conclude that overgrowth of colonies of *lac*-amplified cells by point-mutant cells does not occur as postulated in the sequential, amplification–mutagenesis model (in which one point-mutant cell is proposed to overgrow 10^5^ *lac*-amplified cells; Hendrickson et al. 2002). The only circumstance under which overgrowth might be seen would be if the mutation occurred in the first ten cells of a microcolony. With only approximately 300 *lac* copies present at the ten-cell stage, this mutation frequency, approximately 3 × 10^−3^ mutations per *lac* copy, would be untenably high in any current mutation model.

### Amplification and Not Point Mutation Requires DNA Pol I

**Figure 5:**
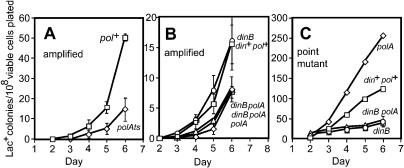
Pol I Is Required for Adaptive Amplification and Not Point Mutation Strains were plated on lactose-minimal medium and Lac^+^ colonies counted daily (see Materials and Methods). The plots are cumulative, showing the mean of 3–4 cultures with one SEM. Strains used: FC40 *dinB^+^ polA^+^* *fadAB*::Tn*10*Kan*,* SMR3490 (squares); FC40 *dinB^+^ polA12*(Ts)*fadAB*::Tn*10*Kan*,* SMR3491 (diamonds); FC40 *dinB10 polA12*(Ts)*fadAB*::Tn*10*Kan*,* PJH308 and PJH309 (triangles, inverted triangles); and FC40 *dinB10 pol^+^ fadAB*::Tn*10*Kan*,* PJH310 (circles). All cultures were grown at 30 °C and the experiments conducted at 37 °C. (A) An example of the effect of *polA*(Ts) on the yield of *lac-*amplified colonies, showing a partial requirement for *polA* at a semi-permissive temperature. (B) and (C) show the effect of the *dinB10* mutation on adaptive *lac-*amplification and point mutation, respectively. The *dinB polA*(Ts) cells display the decreased *lac* amplification of the *polA* mutant (B), and the decreased point mutation characteristic of the *dinB* mutant (C), demonstrating that the decrease in *lac* amplification rate does not result from channeling of *lac*-amplified cells into a point mutation pathway. These data (C) also show that the absence of Pol I increases point mutation (reported previously, Harris 1997) in a completely DinB-dependent manner. This could occur via the absence of Pol I leading to SOS induction (Bates et al. 1989) and more DinB/Pol IV, or via relief of a competition between high-fidelity Pol I and error-prone Pol IV at the replisome. Neither of these ways should affect*lac* amplification, which is Pol IV–independent, as observed (B).

If adaptive amplification were a prerequisite for point mutation, then all genetic requirements for amplification should also be required for point mutation. We report the first genetic requirement found for amplification that is not also required for point mutation: Pol I. Pol I, encoded by *polA,* is required for most amplification (Figure 5A), but adaptive mutation is increased in a *polA* mutant (Figure 5C). We used the temperature-sensitive *polA12* (polA[Ts]) allele incubated at semi-permissive temperature, because *polA* null mutation causes inviability in this strain background (Harris 1997). In repeat experiments, we saw a 2- to 6-fold reduction in amplification rate. In the same experiments, adaptive mutation was unchanged or increased by up to 8-fold.

The possibility that amplification occurs in the Pol I–deficient strain but that the Lac^+^ colonies are unable to grow to visibility in the time-span of an adaptive mutation experiment is ruled out by reconstruction experiments, in which three independent *lac-*amplified isolates carrying *polA*(Ts) or *polA^+^* formed visible colonies in 5.25 ± 0.17 d and 5.39 ± 0.24 d, respectively (mean of three strains ± standard error of the mean [SEM]). Another possibility that we considered is that the *polA* mutation, which causes an increase in SOS induction (Bates et al. 1989), might channel amplified DNA into the point mutation pathway by increasing SOS-induced mutagenesis, so that there is an increase in mutation concomitant with a decrease in amplification. We were unable to test this directly by preventing SOS induction, because the non-inducible allele of *lexA* confers low viability in combination with *polA*(Ts) (data not shown)*.* However we tested this possibility using *dinB*-defective cells. DinB/Pol IV accounts for most, if not all, of the requirement of adaptive mutation for SOS induction (McKenzie et al. 2001). The results are shown in Figure 5 Two independent isolates of the double mutant *dinB polA*(Ts) show the reduction in point mutation (Figure 5C) expected of *dinB* (McKenzie et al. 2001) and the reduction in amplification (Figure 5B) reported above for *polA*(Ts)*.* This demonstrates that the decrease in *lac-*amplified colonies in *polA*(Ts) strains is not due to conversion/channeling of *lac*-amplified cells into point mutants, but rather is caused by reduction of the number of *lac*-amplified cells formed. We conclude that Pol I is required specifically for adaptive amplification and not point mutation. These data are incompatible with models in which amplification is a prerequisite for point mutation, and support models in which point mutation is an independent mechanism.

### Amplification Does Not Induce SOS

**Figure 6:**
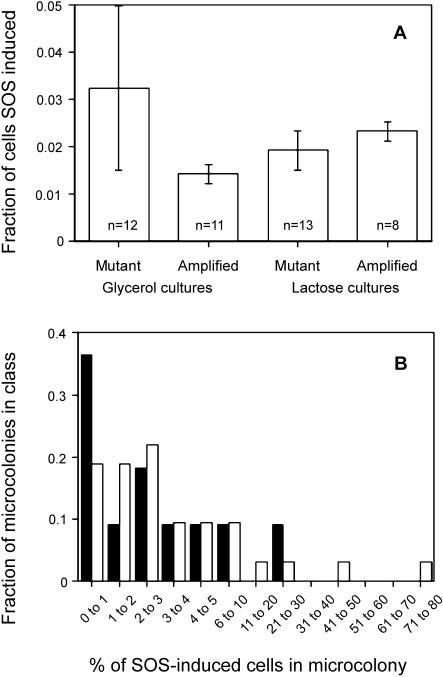
DNA Amplification Does Not Induce the SOS Response (A) Known *lac*-amplified and point-mutant (control) derivatives of SMR6039 were grown in liquid M9 medium containing either lactose or glycerol. Mid-logarithmic phase cells were harvested and scored microscopically for GFP fluorescence, using an Olympus BL51 microscope mounted with a mercury lamp UV source and a High Q Endow GFP emission fluorescence filter cube. Some 1,000–2,000 cells from each of 4–10 fields were scored per determination. Error bars indicate one SEM for 8–13 cultures as indicated. (B) Microcolonies were harvested and suspended in 500 μl of buffer, 50 μl of which was spread on LBH X-gal rif solid medium to determine sectoring in resulting colonies. The remainders were concentrated and examined microscopically for GFP fluorescence, counting 60–400 cells per microcolony. These experiments were plated at very low cell density to avoid significant numbers of background Lac^−^ cells being harvested with the microcolonies. Open bars, fraction of stable Lac^+^ isolates (32 microcolonies); black bars, fraction of sectored isolates (11 microcolonies). The two distributions do not differ (*p* = 0.8 by Student's *t*-test). These experiments were repeated, giving similar results.

A final aspect of the sequential model of Hendrickson et al. (2002) was tested. To explain the high frequency of unselected secondary mutations observed in Lac^+^ adaptive point mutants (Torkelson et al. 1997; Rosche and Foster 1999; Godoy et al. 2000), Hendrickson et al. proposed that amplified DNA itself is sufficient to induce a chronic SOS response leading to increased mutation, and that, therefore, the SOS regulon should be induced in all or most cells carrying amplification. By contrast, hypermutation models (e.g., Rosenberg 2001; Hersh et al. 2004; Rosenberg and Hastings 2004a) suggest that a hypermutable state, possibly an SOS response, is induced only transiently in a subpopulation of starved cells, and that this condition will not persist for long in cells that are able to utilize lactose because they no longer experience the stress of starvation. To determine whether the involvement of SOS in adaptive point mutation (Cairns and Foster 1991; McKenzie et al. 2000) is a consequence of amplification, we constructed a strain in which the green fluorescent protein (GFP) gene is expressed under the control of the SOS-regulated *sulA* promoter (see Figure 1E and 1F). *sulA* is one of about 40 SOS (DNA damage inducible) genes in E. coli (Courcelle et al. 2001). Figure 6A shows that about 2% of cells in the growing cultures showed spontaneous SOS induction, as revealed by GFP fluorescence. There are no detectable differences in this proportion between Lac^+^ point-mutant and *lac*-amplified cultures, or between cultures grown with (lactose medium) or without (glycerol medium) selection for lactose utilization. Control experiments showed that 3 h after exposure to 20 J/m^2^ of ultraviolet light, all cells were induced for SOS (data not shown). Thus, amplification per se does not induce an SOS response.

**Figure 7:**
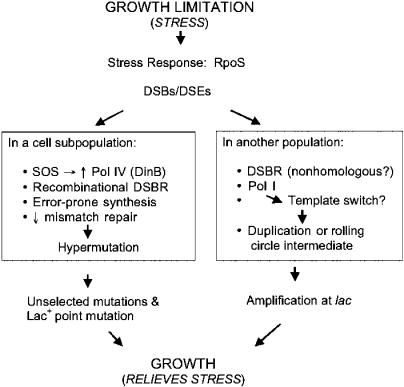
Stress-Response Models for Adaptive Amplification and Point Mutation Modified from Lombardo et al. (2004) and Rosenberg and Hastings (2004a). DSBR, DSB repair; hypermutation, increased mutation at *lac* and elsewhere. The origins of DSEs during starvation on lactose medium in this assay system are unknown, and possibilities are reviewed elsewhere (Rosenberg 2001). On the F′ plasmid carrying the *lac* gene, DSEs may be frequent and may be derived from chronic single-strand nicks at the origin of transfer. However chromosomal mutation during starvation on lactose medium in these cells also requires DSB repair proteins (Bull et al. 2001), and so probably results from (lower levels of) DSEs generated in the chromosome, independently of the transfer origin, or perhaps by transient integration of the F′ into the chromosome (Hfr formation). Both adaptive point mutation and amplification are proposed to be outcomes of RpoS-dependent stress response, which both mechanisms require (Lombardo et al. 2004), and to result from alternative error-prone ways of repairing DSBs.

We also studied the persistence of SOS induction in microcolonies during an adaptive mutation experiment. We isolated microcolonies from lactose plates, spread a sample on LBH (rich) X-gal rifampicin (rif) plates to determine which colonies were point mutant and which *lac*-amplified, and screened other cells from the microcolonies for GFP production. The data presented in Figure 6B show that there is no difference between point-mutant and *lac*-amplified microcolonies in the distribution of cells producing GFP (*p =* 0.8 by Student's *t*-test) and that neither shows persistent SOS induction. Thus, even under adaptive mutation experimental conditions, amplification is not sufficient to induce the SOS regulon. The results also show that SOS induction does not persist for many generations after stress has been released. The data are compatible with hypermutation models for adaptive mutation (Rosenberg 2001; Figure 7), wherein the SOS response is induced transiently during starvation, and are incompatible with the postulate that chronic SOS induction is caused by amplified DNA.

### Further Discussion

#### Amplification is a separate genetic endpoint that allows growth under selection

The experiments reported here were designed to determine whether DNA amplification is an intermediate in the formation of adaptive point mutations in the Lac system, or whether the two are independent end points of genetic change under stress, each of which adapts cells to their environment. All of the data presented support the idea that *lac* amplification and point mutation are alternative end points, either of which allows growth on lactose. These data are reviewed conveniently by contrast with the sequential, amplification–mutagenesis model, as follows.

#### Significance and predictions of amplification–mutagenesis

The experiments reported test the amplification–mutagenesis model for the mechanism of adaptive mutation in the E. coli Lac system (Andersson et al. 1998; Hendrickson et al. 2002). The model's significance lies in its apparent explanation of adaptive mutation phenomenology without invoking an environmentally induced increase in mutation rate, in adherence to conservative neo-Darwinist ideas about constant, gradual evolutionary change (e.g., Mayr 1982). In the model, spontaneous amplification of *lac* allows growth of cells into microcolonies on lactose medium; replication of the multiple *lac* copies generates a point mutant at about the 10^5^-cell stage; and the point-mutant cell overgrows the *lac*-amplified cells, leading to colonies consisting mostly of point-mutant cells with a few (10^−2^–10^−3^) *lac*-amplified cells. This model derives most of its support from the observation of sectored-colony-forming cells in colonies of point-mutant cells (Andersson et al. 1998; Hendrickson et al. 2002).

#### Independent point mutation and amplification mechanisms supported

By contrast, we found, first, that when stringent scoring methods are applied, and all cells in very young microcolonies analyzed, most point-mutant Lac^+^ colonies are not mixtures of stable point-mutant cells with 10^−2^–10^−3^ unstable (amplification bearing) cells, but rather are pure (see Table 1). This was verified with sensitive genetic selection methods that can detect single *lac-*amplified cells among microcolonies (see Table 2), and was found to be true even at the two- to 40-cell stage of colony growth (see Table 3). We conclude that adaptive point mutants arise directly from *lac^−^* single cells without an intermediate stage involving colonies of *lac-*amplified cells, and that the *lac-*amplified cells are a separate adaptive outcome.

Previous observations of mixed colonies (Andersson et al. 1998; Hendrickson et al. 2002) seem likely to have arisen from accidental overlap of point-mutant and Lac^−^ colonies that were not related, which would cause sectored, but not unstable colonies. We observed these, recapitulating the previous results (see Table 1; Figure 1C; Poteete et al. 2002), only when we failed to apply stringent criteria for identification of *lac*-amplified cells. Such colonies might also have arisen from overlap of stable with unrelated unstable microcolonies, which were not screened out stringently (Hendrickson et al. 2002), as done here (see Figure 2; Table 1).

Second, we found that microcolonies smaller than 10^5^ cells are not mostly *lac*-amplified, as the amplification–mutagenesis model demands, but rather that microcolonies carrying amplification are rare initially and frequent later, with kinetics that anticipate the arising of *lac*-amplified visible colonies (See Figure 3). Third, when sampled early and late, young microcolonies with *lac* amplification give rise to *lac*-amplified, not point-mutant, visible colonies (see Figure 4). Fourth, artificial mixtures of point-mutant with *lac*-amplified cells do not show the predicted takeover of the amplification carriers by the point mutants (one point mutant overtaking 10^5^ *lac*-amplified, as predicted Hendrickson et al. [2002] and Roth et al. [2003]) unless the point mutants comprise a very large fraction (10%) of the total (see Table 4). Fifth, we show that DNA Pol I is required for *lac* amplification and not point mutation (see Figure 5), demonstrating that *lac* amplification is not a prerequisite for point mutation. We conclude that amplification is an independent outcome that adapts cells, not a transient intermediate en route to point mutation.

#### Amplification does not induce SOS

To explain the genome-wide hypermutation observed in Lac^+^ point mutants (Torkelson et al. 1997; Rosche and Foster 1999; Godoy et al. 2000) with a sequential model, Hendrickson et al. (2002) suggested that amplified DNA per se induces an SOS response and increased mutability. Using an SOS-inducible *gfp* reporter gene, we find that amplified DNA does not induce SOS (see Figure 6). This conclusion was also supported previously by the observation that cells carrying amplification do not display general hypermutation (Hastings et al. 2000).

#### Apparent support for sequential model compatible with independent pathways

An additional result previously interpreted as support for sequential models was that when a gene counter-selectable at more than one copy was placed next to *lac,* point mutation was decreased (Hendrickson et al. 2002). However, we note that this is predicted by our non-sequential hypermutation model for point mutation (below) as well, in which point mutation is proposed to result from error-prone DNA double-strand-break (DSB) repair, because more than one copy of *lac* must be present to allow repair by homologous recombination. This copy could be on another DNA molecule (a sister chromosome) or the same one (a duplication), but either way it is required for error-prone repair leading to mutation, and its counter-selection would decrease point mutation, even though amplification is not an intermediate in the process.

#### Branched- or independent-pathway, stress-response models for adaptive amplification and point mutation

In Figure 7, we outline a model supported by previous and current data. In this model, amplification and point mutation result from alternative modes of error-prone DNA DSB repair caused as stress responses to starvation. Adaptive point mutation requires homologous recombination proteins, including those specific to DSB repair, implicating DSBs or double-strand ends (DSEs) as a molecular intermediate (Harris et al. 1994). We suggest that adaptive point mutations form by homologous recombinational DSB/DBE repair that is error prone due to use of the SOS-inducible (Kim et al. 1997) and starvation-inducible (Layton and Foster 2003) error-prone DNA polymerase, Pol IV/DinB. The SOS response (Cairns and Foster 1991; McKenzie et al. 2000) and DinB are required for most (85%) adaptive point mutation and not for *lac* amplification in E. coli (McKenzie et al. 2001). Point mutants, but not *lac*-amplified cells (Hastings et al. 2000), carry high levels of mutation of genes throughout their genome (Torkelson et al. 1997; Rosche and Foster 1999; Godoy et al. 2000). Because these levels of mutation are not found in most Lac*^−^* cells starved on the same plates, we infer that only a subpopulation of the starving cells experiences this transient hypermutation (Torkelson et al. 1997), perhaps those that are induced for the SOS response (McKenzie et al. 2000) or the SOS and RpoS stress response (which is required, discussed below) (but see Foster 2004; Rosenberg and Hastings 2004a). Mismatch repair, which corrects replication errors, also becomes limiting specifically during adaptive mutation, leading to increased mutagenesis (Harris et al. 1997); this might occur by titration of mismatch repair proteins by excess DNA polymerase errors (Harris et al. 1997) made by Pol IV (McKenzie et al. 2001). This model, which includes elevated rates of general (not *lac*-specific) genetic change in response to stress, is compatible with Darwinism, which also encompasses changing rates of heritable variations (Darwin 1859), but not with the conservative neo-Darwinist constraint of gradual evolutionary change (e.g., Mayr 1982), a major impetus for the sequential, amplification–mutagenesis model (Andersson et al. 1998; Hendrickson et al. 2002).

#### Amplification mechanism and the role of Pol I

Amplification, on the other hand, could be formed by non-homologous repair of the DSEs (Figure 7), leading to a gene duplication or rolling-circle replication, either of which might amplify rapidly to produce the 20–100 copies of *lac* that allow growth without a point mutation (Hastings and Rosenberg 2002). Alternatively, homologous DSE repair might produce replication forks that lack some of the control of origin-initiated replication and stall in the nucleotide-depleted milieu of starvation. We suggest that template switching can occur at a stalled fork and produce a duplicated-DNA segment or a rolling-circle intermediate from which amplification results. This is similar to template-switching recombination models (Bzymek and Lovett 2001). This template-switching event might occur preferentially with Pol I, which is capable of template switching in vitro (Kornberg and Baker 1992). The partial requirement for Pol I might result from the use of semi-permissive conditions for a conditional mutant. Acquisition of*lac* amplification, and thus the ability to grow on lactose, alleviates the stress and deflects *lac*-amplified cells from the point mutation pathway, making it a (more or less) stable, alternative outcome (Figure 7).

#### There is no appearance of directed mutation in the *lac* system

Unlike the hypermutable state model of Hall (1990), our model (Figure 7) does not specify that cells that do not acquire an adaptive point mutation or amplification die. That feature would be necessary for explaining a lack of mutations in genes other than *lac* (“directed” mutation; Cairns et al. 1988). However, in the E. coli Lac assay, exposure to selection conditions generates (non-adaptive) mutations in other genes efficiently—and by the same recombination-protein- and DinB-dependent mechanism as Lac^+^ adaptive point mutation—both in the F′ that carries *lac* (Foster 1997) and the bacterial chromosome (Bull et al. 2001). A report to the contrary used a different bacterium (Salmonella) with many different genetic features that might not parallel the E. coli Lac assay (Slechta et al. 2002).

#### Adaptive point mutation and amplification in the *lac* system are stress responses

That both adaptive point mutation and amplification are outcomes of a stress response is supported by their requirements for the general-stress-response and stationary-phase transcription factor RpoS (σ^S^) (Lombardo et al. 2004). RpoS upregulates approximately 50 genes in response to starvation, oxidation, pH, heat, and osmotic stresses. RpoS upregulates error-prone Pol IV (Layton and Foster 2003), which might explain its role in the Pol IV–dependent process of adaptive point mutation (McKenzie et al. 2001). However RpoS must play some other role in adaptive amplification, which is Pol IV–independent (McKenzie et al. 2001). RpoS is also required for a Pol IV–independent, starvation-induced hypermutation mechanism in a natural isolate of E. coli (Bjedov et al. 2003) and in other adaptive mutation pathways (Gomez-Gomez et al. 1997; Saumaa et al. 2002), implying that this is a general feature of starvation-induced mutability. Thus, several mechanisms of adaptive genetic change appear to require a differentiated, stress-response condition of the cells, most probably stationary-phase cell physiology. This further supports the idea of stress-induced increases in mutation rate generally.

#### Mutation rates are increased in starving/stressed cells generally

Although it has been argued that increasing mutation generally in response to stress is implausible (Roth et al. 2003), a recent study found that starvation-induced general mutability is the norm in natural isolates of E. coli (Bjedov et al. 2003). The authors found that the vast majority of 787 natural isolates from diverse habitats worldwide produced random, unselected mutations in response to starvation. Multiple mechanisms of mutation in the natural isolates are implied, but at least one of them has similarities to that of the Lac system, and to a different stationary-phase-mutation model system (reviewed by Rosenberg and Hastings 2003), in that these mutation responses require RecA, an SOS-regulated DNA polymerase, and RpoS (Bjedov et al. 2003). Moreover, in the eukaryote *Caenorhabditis elegans,* a new study of mutation (Denver et al. 2004) suggests that cellular stress responses might provoke hypermutation generally, and also lead to a mismatch-repair-compromised transient state (Rosenberg and Hastings 2004c) similar to that suggested here. These systems support the idea that evolution might be hastened during stress. They promise to reveal mutation mechanisms that are likely to pertain to cancer formation and progression, acquisition of drug resistance in pathogens and tumors, and many processes in which clonal expansion under stress or growth limitation follows from an adaptive genetic change.

## Materials and Methods

### 

#### Strains

E. coli strains used are isogenic with FC40 (Cairns and Foster 1991) (also SMR4562, an independent construction of FC40; McKenzie et al. 2000), the *lac*-frameshift-bearing strain in which adaptive mutation and amplification are assayed, and FC29, a non-revertible *lac* deletion strain used to scavenge other carbon sources from the lactose plates (Cairns and Foster 1991). Antibiotic-resistant derivatives SMR4481 (FC40 *malB*::Tn*10*dTet), SMR3598 (FC40 *zff3139*::Tn*10*Kan), SMR533 (FC40 *malB*::Tn*9*), and PJH223, a spontaneous streptomycin-resistant mutant of SMR4562, are resistant to 10 μg/ml tetracycline, 30 μg/ml kanamycin, 25 μg/ml chloramphenicol, and 100 μg/ml streptomycin, respectively. SMR4481 was made by transposition of Tn*10*dTet from lambda NK1323 (Kleckner et al. 1991). SMR533 and SMR3598 were constructed by transduction of FC40 with phage P1 grown on FS2055 (F.W. Stahl, University of Oregon) carrying *malB*::Tn*9,* and STL1605 (S.T. Lovett, Brandeis University) carrying *zff3139*::Tn*10*Kan, respectively. SMR4562 *codA*::*cat* (PJH220) was constructed by linear replacement into SMR4562[pKD46] (plasmid from Datsenko and Wanner 2000) using the *cat* gene from pKRP10 (Reece and Phillips 1995). The isogenic strain pair FC40 *fadAB3165*::Tn*10*Kan (SMR3490) and FC40 *polA12*(Ts)*fadAB3165*::Tn*10*Kan (SMR3491) were constructed by P1 transduction of FC36 (Cairns and Foster 1991) with a P1 lysate carrying *fadAB3165*::Tn*10*Kan from CAG18495 (Singer et al. 1989), or with P1 grown on a strain with *fadAB3165*::Tn*10*Kan from CAG18495 linked with *polA12*(Ts) (Monk and Kinross 1972, via F.W. Stahl, University of Oregon), respectively, followed by transfer of F′128 from FC40 by conjugation. PJH308 and PJH309 were made by P1 transduction of SMR5380 (McKenzie et al. 2001) with lysate from SMR3491 and screened for sensitivity to ultra-violet light (UV) to give two separate UV-sensitive isolates of FC40 *polA*(Ts)*fadAB3165*::Tn*10*Kan*dinB10* and the UV-resistant isogenic control strain PJH310 (FC40 *fadAB3165*::Tn*10*Kan*dinB10*). *polA* mutant strains were grown at 30 °C, and experiments were carried out at 37 °C (a semi-permissive temperature). SMR6039 is a derivative of SMR4562 carrying Δ*attλ*::P*sulAΩgfp-mut2:* the *gfp-mut2* reporter gene (Cormack et al. 1996) under the control of the LexA-regulated *sulA* promoter replacing the phage *attλ* site in the E. coli chromosome (as per Gumbiner-Russo et al. 2001; see below).

#### Construction of an SOS-GFP reporter

The *gfp*-*mut2* gene (Cormack et al. 1996) was inserted into plasmid pACYC184 (New England Biolabs, Beverly, Massachusetts, United States), at the BamHI and HindIII restriction sites, to create pJP21. To fuse the *sulA* promoter to the *gfp* gene, two 5′ phosphorylated oligonucleotides corresponding to the top and bottom DNA fragments of the *sulA −*1 to −67 region were synthesized: BspHI-pSulA-BamHI-top, 5′-P-CATGAGGGTTGATCTTTGTTGTCACTGGATGTACTGTACATCCATACAGTAACTCACAGGGGCTGGATTGATTG-3′ and BspHI-pSulA-BamHI-bot, 5′-P-GATCCAATCAATCCAGCCCCTGTGAGTTACTGTATGGATGTACAGTACATCCAGTGACAACAAAGATCAACCCCT-3′. When these are annealed, a BspHI-complementary end is reconstituted at one end and a BamHI-complementary end at the other. This fragment was ligated into BspHI /BamHI-digested pJP21 to create pJP23. A SphI fragment from pJP23, containing the P*sulA*Ω*gfp-mut2* fusion, was isolated and ligated into SphI-digested pTGV-Light (Gumbiner-Russo et al. 2001) to create pJP30. pJP30 was digested with AscI to generate a linear fragment for chromosomal integration replacing the *att*λ site (Gumbiner-Russo et al. 2001) with the P*sulA*Ω*gfp-mut2* fusion, Δ*att*λ::P*sulA*Ω*gfp-mut2*. Phage P1 grown on this strain was used to construct SMR6039, an SMR4562 derivative carrying Δ*att*λ::P*sulA*Ω*gfp-mut2*, by transduction into SMR5084, genotype SMR4562(λ*xis1 c*I*ts857*Ind), as per (Gumbiner-Russo et al. 2001).

#### Adaptive mutation experiments

Adaptive mutation experiments were performed as per Harris et al. (1996), with the following variations for microcolony experiments: lactose plates were prescavenged by plating 5 × 10^9^ FC29 “scavenger” cells on them and incubating at 37 °C for 24 h, then washing the cells off with M9 buffer; plating of frameshift-bearing cells was not in top agar, but on the plate's surface; growth of Lac^−^ cells throughout the experiments was monitored either by sampling the lawn as described (Cairns and Foster 1991; Harris et al. 1994, 1996) or by microscopic examination. Microscopic examination used 50-fold magnification on a dissecting microscope. The lawn was seen to be smooth at this magnification when less than about 2-fold growth had occurred (number of viable cells relative to starting viable cell measurement). Plates on which growth occurred acquired a punctate appearance. This method was validated by using the two methods in parallel in two experiments, the largest of which involved 24 plates from two cultures. The correlation between growth detected as a doubling in cell number obtained from an agar plug, and the appearance of puncti was complete. No growth (≤ 2-fold) was detected during the relevant times in the experiments reported.

For quantitative measure of mutation and amplification, three or four parallel cultures of each genotype were scored for Lac^+^ colonies each day. Cells from 20 colonies from each culture, or 40 colonies in the case of SMR3491, were plated on LBH rif X-gal to determine the proportion amplified.

#### Whole microcolony analysis

Frameshift-bearing (tester) cells were mixed with 3% each of three or four different derivative testers that carried antibiotic-resistance markers (see Figure 2). Microcolonies were found by use of a dissecting microscope, and harvested on a plug of agar with a capillary tube (see Figure 2). Working at 25× magnification on a dissecting microscope, we were able to find and harvest very young colonies (see Figure 1D), down to a size of 10^2^ cells. The cells were suspended in 500 μl M9 buffer. One percent of the suspension was plated on LBH plates (Torkelson et al. 1997) containing 40 μg/ml X-gal and 100 μg/ml rif (to prevent growth of scavenger cells). The remainder of the suspension was diluted with glycerol (to 12.5%) and stored at −80 °C. Many of the cells in any plating of a harvested microcolony were background (white colony forming) Lac^−^ tester cells that were on the agar plug (see Figure 2). The cells from the microcolony either formed solid blue colonies on LBH X-gal rif plates (see Figure 1A), indicating a point-mutant colony, or sectored blue colonies (see Figure 1B), indicating an unstable *lac*-amplified colony (Hastings et al. 2000). The colonies were scored for sectoring, counted, and patched from the LBH X-gal rif plates onto plates containing antibiotics. Those microcolonies that showed a stable Lac^+^ point-mutant phenotype, had a suitable cell number, and carried one of the minority antibiotic-resistance markers were then plated in toto from the frozen suspension onto LBH X-gal rif medium with the appropriate antibiotic, to search for sectored colonies. Because some sectored colonies are not unstable (e.g., mixtures of stable white and blue colonies that overlap; see Results/Discussion and Table 1), all sectored colonies found were retested by plating a sample on the same LBH X-gal antibiotic medium to determine whether there was continued instability indicative of amplification.

Harvesting the microcolony on a plug of agar with a 25-μl capillary pipette also picks up many Lac^−^ tester cells. The number of these that form colonies during the analysis is reduced by use of antibiotics (as described above) because most cells are not resistant, but the background of tester cells may still be equal in number to those in the microcolony (see Figure 2C). Use of a smaller caliber tube was found to risk losing microcolony cells on the wall of the tube.

#### Selection for *lac*-amplified cells

Microcolonies of PJH220 were harvested as above and suspended in 200 μl of buffer. One microliter (0.5%) of the suspension was spread on LBH X-gal rif medium to determine cell number and which microcolonies were point mutant. Point-mutant microcolonies were then plated to select CamR on LBH plates containing X-gal, rif, and 100 μg/ml chloramphenicol. Plates were scored after 15 to 20 h at 37 °C.

To analyze background cells without microcolonies, plugs of agar were taken from areas of the plates where no microcolony was visible at 20× magnification and the cells suspended in 200 μl of buffer. Half of each suspension was plated on chloramphenicol-containing medium as above, and the other half was plated with scavenger cells onto lactose-minimal medium. Chloramphenicol plates were scored between 15 and 18 h, and lactose plates were scored at 2 d, and again at 7 d. Colonies appearing on lactose medium were scored for amplification by plating a sample of the cells on LBH X-gal rif medium. Colonies appearing on chloramphenicol-containing medium were scored for *lac* amplification (blue and white sectoring) directly on that plate. All CamR colonies were seen to be *lac*-amplified.

The fraction of *lac*-amplified cells detectable by CamR selection was determined as follows. There are two reasons why *lac* amplification might fail to be detected with the CamR selection: first, because some *lac* amplifications have not co-amplified the neighboring *codA*::*cat* gene, and, second, because some *cat-*amplified microcolonies obtained from the lactose plates (grown selectively for Lac^+^ and not for CamR) might have too few *cat* copies for growth on 100 μg/ml chloramphenicol. We determined each of these separately. We found that 59 of 77 *lac*-amplified microcolonies were CamR (at least 77% had co-amplified *cat*), and that 84% of cells from CamR (*cat*-amplified) isolates grown on lactose without chloramphenicol were then able to form colonies on chloramphenicol medium. The cells that did not form colonies on chloramphenicol presumably had lost or reduced their amplification of *cat* and *lac.* This would indicate that 77% of 84% (65%) of all *lac*-amplified cells should be detected by the CamR selection. An independent determination of the number of *cat*-amplified cells that grow on chloramphenicol after growth on lactose was obtained in a reconstruction experiment in which a few CamR cells were plated with a 10^4^-fold excess of Lac^+^ point-mutant cells, mimicking more closely the conditions of the experiments in which microcolonies are obtained. A total of 79% of the *cat-*amplified cells formed colonies on chloramphenicol medium, so 77% of 79% (61%) of all *lac*-amplified cells should be detected. The selection for CamR is actually a more sensitive assay for *lac* amplification than is selection for Lac^+^, as shown by the fact that only 60% of cells from the cultures that showed CamR were able to form colonies on lactose medium under the conditions of an adaptive mutation experiment, though all carried*lac* amplification (indicating that 40% had too few *lac* copies for growth on lactose), whereas 79% were detected on chloramphenicol medium.

#### Colony-fate experiments

Microcolonies were touched with the point of a needle, and cells were transferred to LBH X-gal rif plates, where they were spread by streaking for scoring. Control experiments (not shown) showed that this procedure removed, on average, 5% of the microcolony from the plate. About 50 separate colonies from each microcolony were scored for sectoring. The remainder of the colony was left for 1 to 4 d to form a visible colony in situ. The visible colony was then sampled, plated, and scored for sectoring (see “whole microcolony analysis” above).

#### Competition experiments

M9 lactose liquid cultures of Lac^+^ point-mutant and *lac*-amplified cells were grown (separately) to saturation, then mixed in various proportions, and 0.5 μl of the mixed cell suspensions was put onto the surface of prescavenged lactose plates and grown into visible colonies. These “pseudocolonies” were suspended, and cells were spread on LBH X-gal rif plates at 100 to 200 cells per plate and scored for sectoring blue phenotypes after 2 d growth.

## Supporting Information

### Accession Numbers

The Swiss-Prot ( http://www.ebi.ac.uk/swissprot/) and Ecogene ( http://bmb.med.miami.edu/EcoGene/EcoWeb/) accession numbers for the genes and gene products discussed in this paper are *cat* (Swiss-Prot P62577), *codA* (Swiss-Prot P25524), DNA Pol I (Swiss-Prot P00582), DNA Pol IV/*dinB* (Swiss-Prot Q47155), *fadAB* (Swiss-Prot P21177), *malB (*Swiss-Prot P02943), *gfp-mut2* (Swiss-Prot P42212), *lac* (Swiss-Prot P00722 and P03023), *recA* (Swiss-Prot P03017), *recBC* (Swiss-Prot P08394 and EcoGene EG10825), *rpoS* (Swiss-Prot P13445), *ruvA* (Ecogene EG10923), *ruvB* (Ecogene EG10924), *ruvC* (Ecogene EG10925), and *sulA* (Swiss-Prot P08846).

## Abbreviations

CamR: chloramphenicol resistant/resistance
*cat*: chloramphenicol acetyl transferase gene
cfu: colony-forming units
DSB: double-strand break
DSE: double-strand end
GFP: green fluorescent protein
LBH: Luria-Bertani-Herskowitz medium
Pol: polymerase
polA(Ts): temperature-sensitive *polA12*
rif: rifampicin
SEM: standard error of the mean
UV: ultra-violet light
X-gal: 5-bromo-4-chloro-3-indoyl β-D-galactoside
